# Advancing clinical oncology through genome biology and technology

**DOI:** 10.1186/s13059-014-0427-x

**Published:** 2014-08-07

**Authors:** Anna M Varghese, Michael F Berger

**Affiliations:** Department of Medicine, Memorial Sloan Kettering Cancer Center, New York, NY 10065 USA; Department of Pathology, Memorial Sloan Kettering Cancer Center, New York, NY 10065 USA; Human Oncology and Pathogenesis Program, Memorial Sloan Kettering Cancer Center, New York, NY 10065 USA; Center for Molecular Oncology, Memorial Sloan Kettering Cancer Center, New York, NY 10065 USA

## Abstract

The use of genomic technologies for the molecular characterization of tumors has propelled our understanding of cancer biology and is transforming the way patients with cancer are diagnosed and treated.

## Clinical oncology - facing up to the next steps

More than any other field of medicine, oncology has benefited from recent revolutionary advances in nucleic acid sequencing technology and genomic analysis. For increasingly lower costs and turnaround times, one can profile the full spectrum of genomic alterations in a tumor sample, including sequence mutations, copy number changes and structural rearrangements [1]. Research initiatives have capitalized on the availability of large numbers of tumors in order to delineate the most frequently mutated genes and pathways in a range of cancer types. Collectively these projects, including The Cancer Genome Atlas (TCGA) and the International Cancer Genome Consortium (ICGC), are characterizing the genomes and epigenomes of virtually all common tumor types and producing a more complete understanding of the biology of cancer [2-12]. Just as significantly, the introduction of genomic technologies is transforming clinical practice. Massively parallel ‘next-generation sequencing’ (NGS) has proven to be a powerful molecular-diagnostic tool, enabling the identification of prognostic and predictive biomarkers in individual clinical specimens. Challenges for the widespread implementation of clinical NGS platforms remain, including technical, operational, medical and societal considerations. However, by confronting these issues collectively, we can overcome these challenges and realize the maximum benefit to clinical oncology.

## Opportunities for applying genomics to oncology

Research initiatives such as TCGA and ICGC have led to an improved understanding of cancer biology through the genomic analysis of tumors procured from anonymized patients possessing many different types of cancer. A major goal of these and similar efforts is to identify genes and pathways important to cancer progression in order to design better therapies and interventions. As an early example, the identification of recurrent somatic mutations in the serine/threonine-protein kinase B-raf (*BRAF*) gene from systematic gene sequencing [13] prompted the development of multiple targeted inhibitors of BRAF (notably vemurafenib and dabrafenib), currently approved by the Food and Drug Administration (FDA) for melanoma and showing promise in a range of other cancer types [14,15]. Yet the most exciting and transformative applications of genomics in oncology involve the use of genomic techniques to analyze clinical tumor specimens. First, by sequencing tumors obtained from patients treated with approved and experimental targeted therapies, one can discover clinical biomarkers that predict outcomes and therapeutic response. Second, by prospectively sequencing patients’ tumors as part of their care, one can select personalized therapies to employ based on the individual molecular profile of the tumors. We discuss both of these opportunities below.

### Phenotype to genotype: retrospective sequencing for biomarker discovery

Genomic alterations that predict the likelihood of response to therapeutic agents, especially novel targeted therapies, serve as powerful biomarkers and major determinants of treatment decisions. In many cases, the retrospective characterization of tumors procured from patients with documented clinical outcomes has revealed the molecular basis of drug response. For example, the discovery of epidermal growth factor receptor (EGFR) mutations in lung cancer was prompted by the clinical observation that a subset of patients exhibited a major response when administered tyrosine kinase inhibitors in clinical trials [16–18]. Today, a growing number of clinical trials in oncology are designed with correlative sequence analysis for biomarker discovery written into the study.

Recently, the analysis of ‘exceptional responders’ - cancer patients with an unexpected complete and/or durable response to therapy - has proven to be particularly effective. Studying exceptional responders can reveal specific genomic alterations accounting for their exquisite sensitivity to certain drugs. One can hypothesize that other patients whose tumors bear similar alterations will benefit from the same drugs. Recent successful applications of whole-genome or exome sequencing in exceptional responders include the identification of tuberous sclerosis 1 protein/hamartin (*TSC1*) and serine/threonine-protein kinase mTOR/mammalian target of rapamycin (*MTOR*) mutations in bladder cancer patients responding to everolimus and a mutation in serine/threonine-protein kinase A-Raf (*ARAF*) in a lung cancer patient responding to sorafenib [19–21]. Owing to the promise of this approach, the US National Cancer Institute has launched a program to collect tissue samples and clinical data from up to 200 exceptional responders in an attempt to explain isolated responses to drugs that otherwise failed clinical trials [22].

Retrospective analysis of clinical tumor specimens can also reveal mechanisms of acquired resistance to targeted therapies. By collecting and comparing samples before treatment and at progression, one can discover genetic alterations that emerge during drug exposure and confer drug resistance. This approach has led to the identification of a broad spectrum of resistance mechanisms arising during inhibition of EGFR in lung cancer and during inhibition of BRAF in melanoma [23–27]. Other examples include single-nucleotide mutations conferring resistance to imatinib in leukemia [28], enzalutamide in prostate cancer [29] and anti-estrogen therapy in breast cancer [30,31]. Resistance mutations might be second hits to the drug target itself, could affect downstream genes that reactivate the targeted pathway or might activate alternative pathways that bypass or counteract the effects of the drug. The identification of such mutations in tumors can point to novel combination strategies and/or lead to the development of new, more potent drugs.

### Genotype to phenotype: prospective sequencing for clinical diagnosis

Based in part on the identification of predictive clinical biomarkers from retrospective analyses, there are several tumor types for which prospective mutation profiling as a diagnostic tool is now a standard of care. Patients with metastatic non-small cell lung cancer are routinely tested for *EGFR* mutations and rearrangements of the gene encoding the ALK tyrosine kinase receptor/anaplastic lymphoma kinase (*ALK*) to guide treatment. If a sensitizing mutation in *EGFR* or a rearrangement in *ALK* is identified, treatment with an inhibitor of EGFR or ALK is recommended. While drugs targeting these alterations are FDA approved, several targeted agents have demonstrated activity against lung cancers harboring other genetic alterations. For instance, cabozantinib has demonstrated activity in lung cancers harboring rearrangements in the gene encoding the tyrosine-protein kinase receptor Ret (*RET*), and crizotinib has demonstrated activity in lung cancers harboring amplifications in the gene encoding the hepatocyte growth factor receptor (*MET*) or rearrangements in the *ROS1* gene encoding tyrosine-protein kinase ROS [32–34]. For patients with metastatic colorectal cancer, testing for the presence or absence of hotspot mutations in the genes encoding the GTPases *KRAS* and *NRAS* is recommended to assess whether patients might benefit from EGFR-directed monoclonal antibody therapies utilizing the drugs cetuximab or panitumumab. For patients with melanoma harboring V600 mutations in BRAF, treatment with vemurafenib or dabrafenib is recommended.

Molecular-diagnostics labs have traditionally relied on low-throughput, mutation-specific methods for DNA profiling in patients because there were so few actionable genetic alterations that altered treatment decisions in the clinical care of patients. However, given the growing number of biomarkers and clinical trials studying targeted agents, the approach of testing one mutation at a time is unsustainable. NGS-based assays are replacing these more-focused tests in both academic and commercial settings. The benefits of this are obvious. First, a single NGS test can encompass all ‘actionable’ targets, eliminating the need for multiple parallel tests for different mutations and enabling more-efficient workflows and tissue utilization. Second, the entire coding sequence of target genes can be assayed (rather than only pre-specified sites), facilitating the detection of both common and rare mutations in oncogenes and tumor-suppressor genes. Third, NGS enables the detection of additional classes of genomic alterations, such as copy number changes and structural rearrangements. Finally, subclonal events in heterogeneous tumors can be detected more reliably owing to the high sensitivity afforded by NGS.

Some academic centers have implemented pilot programs for the comprehensive genomic characterization of tumors from selected patients by means of whole-genome or exome sequencing (DNA-Seq) and transcriptome sequencing (RNA-Seq) [35,36]. Through expert review and curation of these expansive data sets, clinically relevant alterations can often be identified that direct treatment with rationally chosen available therapies. ‘Genomic tumor boards’ composed of clinicians and scientists trained in medical oncology, cell biology, genomics and bioinformatics, as pioneered by the University of Michigan and elsewhere, are forming at many leading cancer centers to review and interpret clinical genomic data in order to recommend and guide therapy [35]. These organizations also serve to educate members of the medical community as to the power and intricacies of genomic analysis and are catalyzing the development of communal frameworks for the clinical annotation and interpretation of somatic alterations.

Owing to practical barriers in the implementation of this comprehensive approach for all patients, namely its high cost and low throughput, large-volume molecular-diagnostics labs have focused instead on targeted sequencing of key cancer-associated genes as a feasible and economical alternative [37–42]. Furthermore, the deeper sequence coverage afforded by targeted sequencing enables low-allele-frequency mutations in heterogeneous or low-purity tumors to be detected with greater sensitivity. Multiplexing through the use of sample ‘barcodes’ permits many tumors to be profiled in a single NGS run [43]. This has enormous implications for the design and implementation of clinical trials in oncology. By systematically screening large numbers of patients with metastatic disease for common and rare ‘druggable’ mutations, patients can be pre-identified for future trials involving the most promising targeted therapies, and new trials can rapidly accrue patients with the greatest likelihood of exhibiting a clinical response. Novel clinical trial designs have emerged as a direct result of advances in molecular profiling. One such trial, often called a ‘basket’ study, involves a single targeted drug administered to patients across many different tumor types (baskets) that share a common genomic profile. Other clinical protocols utilize centralized NGS-based diagnostic testing to assign patients recruited through cooperative groups to multiple separate trials involving targeted agents, pioneered by the NCI-MATCH study and others [22].

## Challenges in applying genomics to oncology

In order for genomic insights and technologies to be truly transforming in oncology, we must develop and implement high-throughput molecular-diagnostic tests that exhibit both clinical validity and clinical utility. Assays must achieve rapid turnaround times at low cost, encompass all classes of sequence-based mutations and structural alterations, and operate on small biopsies, formalin-fixed paraffin embedded (FFPE) tissue and cytological specimens [44]. Furthermore, results must be annotated with associated genomic and clinical data pertaining to the specific set of mutations observed in each individual tumor in order for oncologists to make the most-informed treatment decisions. This endeavor, although very promising, is fraught with challenges.

### Technical and operational considerations

The development of assays compatible with low-quality specimens and low input DNA amounts remains a technical challenge. For these suboptimal samples that are routinely encountered in clinical settings, comprehensive genomic analysis is not always possible. PCR-based capture technologies can produce deep-coverage sequence data with very little DNA, but targets are generally small, and the detection of copy-number alterations and structural rearrangements is compromised. With slightly more DNA, hybridization-based capture assays enable an expanded number of target genes and alteration types but still can exclude important regions of the genome. Even whole-exome sequencing, encompassing all protein-coding genes in the genome, will miss many structural alterations as well as nucleotide substitutions involving regulatory regions such as the promoter of the gene encoding telomerase reverse transcriptase (*TERT*), which rank among the most frequently observed mutations in all cancers [45–47]. Individual clinical labs are left to choose how many (and which particular) genes to sequence based largely on their anticipated volume and diversity of cases, desired turnaround time and cost, and bioinformatics capabilities.

Indeed, providing support for bioinformatics represents one of the most significant challenges for the widespread implementation of clinical NGS workflows. Historically, pathology departments at hospitals and academic cancer centers have not employed large numbers of bioinformaticians. As a result, the recruitment and training of capable computational staff is of utmost importance in order to develop, maintain and deploy pipelines for the analysis of clinical cancer genomic data. The bioinformatics algorithms and software that comprise these pipelines are continually evolving, with new tools emerging constantly, making it difficult to standardize analysis procedures. Additionally, issues pertaining to the management and storage of large data files and the establishment of high-performance computing infrastructures for data processing and analysis represent new challenges for clinical labs and departments. The complexity of most hospital information systems could further hinder efforts to deposit molecular-diagnostic results into a patient’s electronic health record and to link genomic data to other clinical, phenotypic and demographic data. This integration of genomic and clinical data is crucial for the long-term goal of achieving better outcomes for patients with cancer.

In order for clinical NGS results to be used by oncologists to influence treatment decisions, tests must be performed in Clinical Laboratory Improvement Amendments (CLIA)-compliant laboratories, governed by the Centers for Medicare & Medicaid Services (CMS). Accordingly, extensive documentation and technical validation of sensitivity, accuracy and reproducibility are required before any genomic assay can be prospectively administered and/or billed to healthcare payers. While some institutions have initiated programs where large-scale genomic analysis is performed in research labs, followed by confirmatory testing in CLIA-compliant labs, this approach is unsustainable when NGS-related costs cannot be recovered through reimbursement.

### Clinical considerations

While use of comprehensive molecular profiling has produced success stories, such as the remarkable responses to erlotinib for patients with EGFR-mutant lung cancers or crizotinib for patients with ALK-positive lung cancers, these findings have also resulted in challenges and questions concerning how to proceed.

First, comprehensive molecular profiling using any of the assays or technologies described above will yield information about both well-known molecular drivers in cancer and countless more alterations whose biological and clinical significance is unclear. It is often difficult for a clinician to make the crucially important distinction between key ‘driver alterations’ that should impact treatment and less-relevant ‘passenger mutations’. Some publicly available knowledge banks have been created to help doctors and patients interpret the significance of many commonly seen alterations, such as My Cancer Genome developed at the Vanderbilt-Ingram Cancer Center. While these sites are excellent resources to learn about clinically and pathologically annotated alterations, they are not comprehensive and cannot be expected to include information on all possible alterations that a clinician could encounter. Similarly, there is little guidance for the management of patients with multiple driver alterations in more than one gene. One might rationally assume to administer combinations of targeted therapies; however, without knowing the toxicity profiles or optimal dosing and scheduling of the combination, this strategy is problematic.

Also, the use of targeted therapies in the treatment of cancer is rarely straightforward, even in the presence of individual well-characterized driver alterations. NGS testing of heterogeneous tumors can reveal targetable mutations at subclonal allele frequencies, the clinical consequences of which are uncertain. Furthermore, the biological and clinical context can be extremely important. For instance, while patients with melanomas harboring BRAF V600E mutations almost always respond to inhibitors of BRAF, this direct treatment approach has not been replicated for patients with BRAF-mutant colon cancers. Colon cancers harboring BRAF V600E mutations do not respond to single-agent vemurafenib - this is thought to be related to feedback reactivation of alternative or upstream signaling pathways [48,49]. Studies are ongoing to explore and exploit these mechanisms by using combination therapy in the treatment of BRAF-mutant colon cancer. In order for clinicians to make informed treatment decisions, molecular-diagnostic reports must display alongside each mutation disease-specific contextual annotations in a succinct and easily digestible form.

Which specimens to analyze and when to analyze them are also questions that arise in the clinical care of patients. Given emerging data about tumor heterogeneity in metastatic disease, it is unclear that a single biopsy of a single site of metastatic disease will accurately capture driver alterations that would most impact a patient’s clinical care. Additionally, it is unclear that biopsies obtained at the time of diagnosis remain relevant after patients have developed acquired resistance to targeted therapies or have developed recurrent disease after initial therapies for early-stage cancer. While biopsies taken at the time of acquired resistance are becoming standard in many clinical trials of targeted agents, these biopsies are not routinely performed in the clinical care of patients.

Most importantly, targeted therapies are not curing patients of their cancer. While targeted therapies have yielded promising, dramatic and life-changing responses for many patients with cancer, the reality is that these responses are generally short lived. For instance, the average responses to erlotinib among patients with EGFR-mutant lung cancer and vemurafenib for patients with BRAF V600E mutant melanoma are 11 months and 5 months, respectively [14,50]. All of these patients will ultimately acquire resistance and succumb to their disease. In fact, some have questioned the cost effectiveness of targeted therapies, given the rare frequency of some driver mutations and the high cost of these therapies [51].

### Societal considerations

The societal and ethical implications of comprehensive genetic testing must also be considered in this rapidly changing technological landscape. While focused diagnostic tests including targeted ‘hotspot’ panels utilize only tumor-derived DNA, more-comprehensive NGS approaches typically require germline DNA to distinguish between novel somatic mutations and inherited variants. The use of germline DNA poses the risk that incidental findings could be revealed relating to inherited susceptibility to cancer or other diseases. This has led institutions to consider different strategies of informed consent and pre-test genetic counseling. However, given the time pressures of clinical care, the ability to perform thorough genetic counseling in a routine fashion is limited. For those patients in whom an inherited predisposition to cancer is discovered, care must be taken to ensure the patients’ autonomy and privacy, protect them and their families from possible discrimination and also manage the unintended emotional and psychological consequences that such a diagnosis brings.

Additionally, it remains unclear whether NGS-based molecular profiling is cost-effective or even clinically effective in the care of patients outside of lung cancer, melanoma and colon cancer. As a result, insurance companies have demonstrated a reluctance to reimburse the cost of comprehensive testing in many tumor types. While large academic cancer centers might be able to offset costs temporarily through grants and philanthropic contributions, broad access to these tests for patients in the community has not been achieved. Demonstrating the general clinical utility of NGS-based molecular profiling is essential in this regard. A related hindrance is that, when actionable mutations are detected in unexpected tumor types, insurance companies are often unwilling to reimburse the off-label administration of therapies approved in other diseases.

## Concluding remarks

While genomic knowledge has been successfully applied to direct clinical decisions in several cases, the implementation of clinical genomic workflows for oncology has proven to be complex. Nevertheless, the introduction of NGS technology has the potential to transform clinical oncology, and it is incumbent upon clinicians, scientists, regulators, payers and patients to work collectively to overcome the obstacles that stand in its path.

## Abbreviations

CLIA: Clinical Laboratory Improvement Amendments
CMS: Centers for Medicare & Medicaid Services
FDA: Food and Drug Administration
FFPE: Formalin-fixed paraffin embedded
ICGC: International Cancer Genome Consortium
NGS: Next-generation sequencing
TCGA: The Cancer Genome Atlas
